# Cyclic processes in the uterine tubes, endometrium, myometrium, and cervix: pathways and perturbations

**DOI:** 10.1093/molehr/gaad012

**Published:** 2023-05-24

**Authors:** Sarah J Holdsworth-Carson, Ellen Menkhorst, Jacqueline A Maybin, Anna King, Jane E Girling

**Affiliations:** Julia Argyrou Endometriosis Centre, Epworth HealthCare, Melbourne, Australia; Department of Obstetrics and Gynaecology, University of Melbourne and Gynaecology Research Centre, Royal Women’s Hospital, Melbourne, Australia; Department of Obstetrics and Gynaecology, University of Melbourne and Gynaecology Research Centre, Royal Women’s Hospital, Melbourne, Australia; Institute for Regeneration and Repair, MRC Centre for Reproductive Health, University of Edinburgh, Edinburgh, UK; Department of Obstetrics and Gynaecology, NHS Lothian, Edinburgh, UK; Department of Obstetrics and Gynaecology, University of Melbourne and Gynaecology Research Centre, Royal Women’s Hospital, Melbourne, Australia; Department of Anatomy, School of Biomedical Sciences, University of Otago, Dunedin, New Zealand

**Keywords:** menstrual cycle, endometrium, myometrium, cervix, uterine tube, fertility, fallopian tube, menstruation

## Abstract

This review leads the 2023 Call for Papers in MHR: ‘Cyclical function of the female reproductive tract’ and will outline the complex and fascinating changes that take place in the reproductive tract during the menstrual cycle. We will also explore associated reproductive tract abnormalities that impact or are impacted by the menstrual cycle. Between menarche and menopause, women and people who menstruate living in high-income countries can expect to experience ∼450 menstrual cycles. The primary function of the menstrual cycle is to prepare the reproductive system for pregnancy in the event of fertilization. In the absence of pregnancy, ovarian hormone levels fall, triggering the end of the menstrual cycle and onset of menstruation. We have chosen to exclude the ovaries and focus on the other structures that make up the reproductive tract: uterine tubes, endometrium, myometrium, and cervix, which also functionally change in response to fluctuations in ovarian hormone production across the menstrual cycle. This inaugural paper for the 2023 MHR special collection will discuss our current understanding of the normal physiological processes involved in uterine cyclicity (limited specifically to the uterine tubes, endometrium, myometrium, and cervix) in humans, and other mammals where relevant. We will emphasize where knowledge gaps exist and highlight the impact that reproductive tract and uterine cycle perturbations have on health and fertility.

## Introduction

The menstrual cycle encompasses the series of changes in hormone production that result in alteration of the structure and function of the female reproductive tract, including the uterine tubes (oviduct or fallopian tube), uterus, and uterine cervix. Women and others who menstruate in high-income countries can expect to experience ∼450 menstrual cycles in their lifetime (reviewed by Eaton *et al.* (1994) and Critchley *et al.* (2020a)). The first occurrence of menstruation is termed menarche and the average age of menarche in high-income countries is 12.5 years (Morris *et al.*, 2011; Colich *et al.*, 2020; Martinez, 2020). At the other end of the reproductive lifespan, menopause is defined as the permanent cessation of menstruation resulting from the loss of ovarian follicular activity. Natural menopause is recognized to have occurred after 12 consecutive months of amenorrhoea, for which there is no other obvious pathological or physiological cause. The average age of menopause in high-income countries is 50.5 years, but this is affected by demographic, reproductive, genetic, and environmental factors (Gold, 2011).

**Figure 1.**
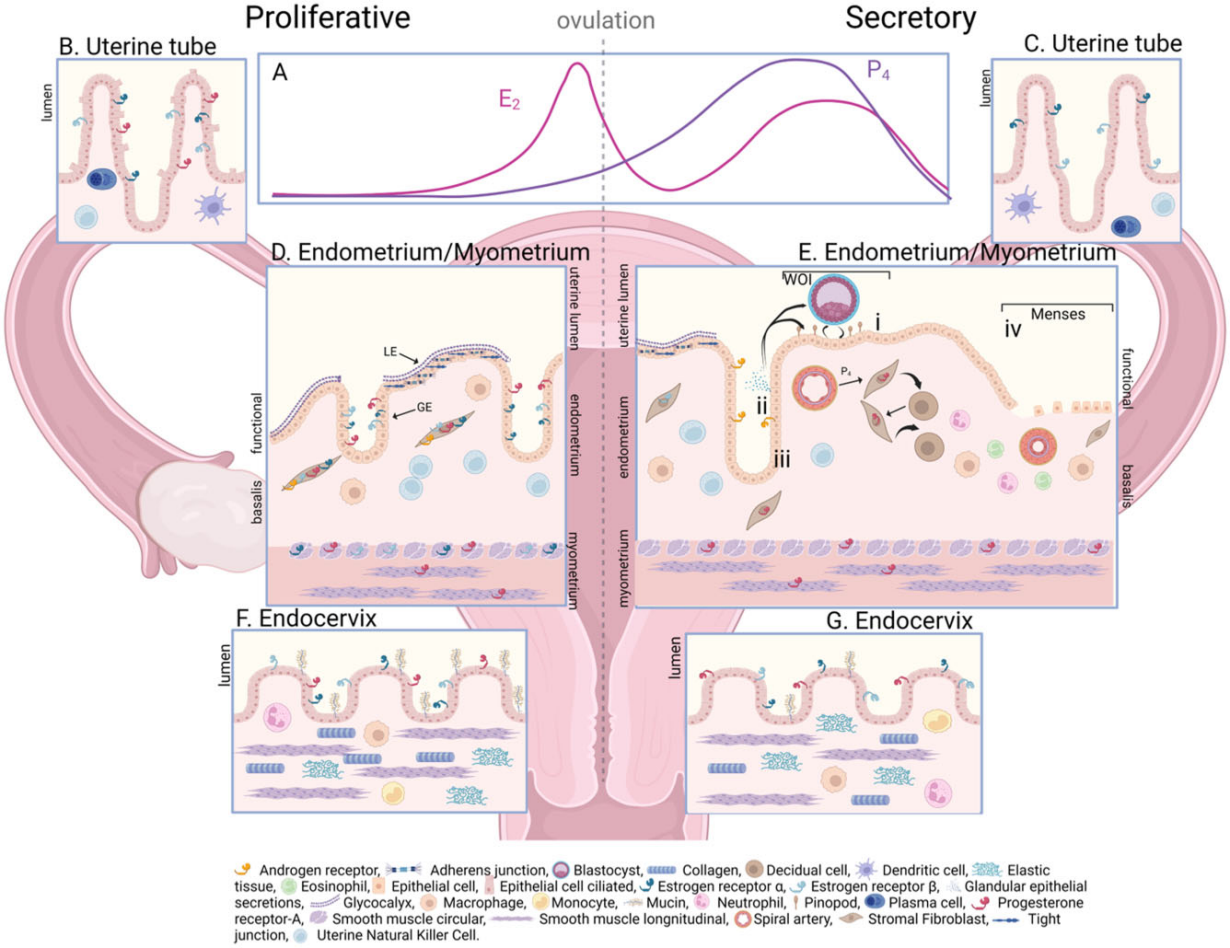
**Cyclical ovarian hormone production and changes in uterine tube, endometrial, myometrial, and endocervical tissue composition and structure during the menstrual cycle.** (**A**) During the proliferative phase oestradiol (E_2_) is the dominant ovarian hormone. Following ovulation, the corpus luteum secretes high levels of progesterone (P) which subsequently fall in the absence of pregnancy, triggering the end of the menstrual cycle and onset of menstruation. A single columnar epithelium lines the uterine tube mucosa (**B** and **C**) which contains lymphoid and myeloid cells, including uterine natural killer (uNK), plasma, and dendritic cells. During the proliferative phase (B), epithelial cells express estrogen receptors (ER)-α and -β and progesterone receptor (PR)-A and E_2_ induces increased epithelial cell height and cilia activity. In the secretory phase, epithelial PR-A is reduced and E_2_ induced epithelial changes are lost. The uterine lumen (**D** and **E**) is lined by the endometrial epithelial luminal epithelium (LE) covering the multi-cellular stromal compartment of the endometrium which is internal to the muscular myometrium. The glandular epithelium (GE) invaginates from the LE into the endometrial stroma. During the proliferative (D) phase, E_2_ induces endometrial cell (GE and stroma) proliferation via ERα and ERβ. ER expression in the myometrial myocytes is maximal during the proliferative phase. PR-A expression in myocytes is consistent throughout the menstrual cycle. Androgen receptors (AR) are also present in stromal fibroblasts during the proliferative phase. During the secretory phase (E), ER decline in the glandular epithelium and stroma, although ERβ levels are maintained in the stroma. During the period of endometrial receptivity (window of implantation, WOI), the endometrium undergoes significant transformation: (i) the luminal epithelium undergoes epithelial–mesenchymal transition, including loss of tight junctions and adherens junctions, with enzymatic clearance of the glycocalyx and the appearance of pinopods; (ii) the glandular epithelium becomes highly secretory, with released factors acting on the luminal epithelium or blastocyst to promote implantation; and (iii) stromal cells in the endometrium undergo ‘decidualization’ in response to P_4_ and factors released from decidualized stromal cells. In the absence of P_4_ and E_2_, menses (iv) and endometrial breakdown is initiated. There is an influx of immune cells into the endometrial stroma, vasoconstriction of spiral arteries, and breakdown and shedding of the functional layer of the endometrium. The endocervix joins the vagina to the uterus and is comprised of mucin-secreting columnar epithelium overlaying a dense fibro-muscular stroma made up of connective and elastic tissues and smooth muscle. During the proliferative phase (**F**), mucin secretion increases with rising E_2_ and infiltrating immune cell numbers drop. During the secretory phase (**G**), mucin secretion reduces with increasing P_4_ and infiltrating immune cell numbers increase. Figure created with BioRender.com.

The primary function of the menstrual cycle is to prepare the reproductive system for pregnancy should fertilization occur. The first day of menstrual bleeding is designated as Day 1 of the menstrual cycle and the typical length of a cycle is 24–38 days (Munro *et al.*, 2018). During the first part of the menstrual cycle, the follicular or menstrual/proliferative phase, oestradiol (E_2_) is the dominant ovarian hormone (Fig. 1). Following ovulation, the corpus luteum secretes high levels of progesterone (P_4_) during the luteal/secretory phase to decidualize and prepare the endometrium for possible implantation (Fig. 1). The corpus luteum also secretes relaxin in the latter half of the secretory phase; its levels are otherwise low or undetectable in humans during the other phases of the cycle (Marshall *et al.*, 2017). In the absence of pregnancy, the ovarian hormone levels fall, with the fall in P_4_ triggering the end of the menstrual cycle and onset of menstruation (Fig. 1). The changes in uterine tubal, endometrial, myometrial, and cervical structure and function in response to fluctuations in ovarian hormone production are the subject of this review. Here, we cover the complex, highly coordinated processes of vascular function, inflammation, and tissue remodelling that occur throughout the menstrual cycle in the reproductive tract. Current understanding of the physiology of the menstrual cycle will be reviewed before examination of the pathologies caused, linked, or exacerbated by cyclical pathways. We hope this review will not only illustrate the current knowledge of the functional and mechanistic pathways related to the reproductive tract and menstrual cycle but also highlight the historical lack of research focus in this area, particularly with respect to the uterine tube, myometrium, and cervix. Our review is the first paper in the call for the 2023 special MHR collection ‘Cyclical function of the female reproductive tract’; we hope that it will attract novel and much needed research papers and provide a platform to facilitate increased awareness of the research need.

## Normal cyclic pathways

### Uterine tube

The uterine tubes, also commonly referred to as fallopian tubes (we will use uterine tubes throughout this article to avoid eponymous language), oviducts or salpinges (Fig. 1), function to transport the fertilized oocyte (zygote) encased in its protective zona pellucida to the uterine cavity for implantation. The tube is comprised of an outer serosa, with inner layers of smooth muscle arranged in a longitudinal and circular manner. Internal to the muscular layers is a mucosa containing lymphoid and myeloid cells. A single layer of columnar epithelium lines the luminal portion of the tube (Horne and Critchley, 2012). Most laterally, the funnel-shaped infundibulum is bordered by fimbriae; these are finger-like projections that aid the movement of the ovulated oocyte into the ampulla, which forms the mid-section of the uterine tube, and then more medially the isthmus, which opens into the uterine lumen. Fertilization takes place in the uterine tube within 24–48 h of ovulation.

The mucosa of the uterine tube is exquisitely sensitive to changes in circulating levels of steroid hormones, particularly E_2_ and P_4_, via cognate nuclear and membrane receptors. In the uterine tube, progesterone receptor (PR) is reduced during the secretory phase but expression of the oestrogen receptors (ERα and ERβ) is maintained (Horne *et al.*, 2009). The cyclic changes taking place (Fig. 1) are necessary to facilitate the transport of spermatozoa to the site of fertilization, whether that be the ampulla as commonly ascribed, or the peritoneum as has also been postulated (Thunnissen *et al.*, 2021). The effects of E_2_, which are subsequently opposed by P_4_, include increased epithelial cell height and cilial activity, protein production and secretory activity, as well as transudation of fluid from capillaries and increased smooth muscle cell activity (Barton *et al.*, 2020). These changes are responsible for production of tubal fluid and embryotropic factors (e.g. antioxidants or growth factors that are produced by the cells lining the uterine tube) needed to facilitate early conceptus development and movement of the conceptus into the uterus.

### Endometrium

The human uterus has three layers: a thin outer serosal layer (perimetrium), a middle smooth muscle layer of myometrium, and an endometrial layer that lines the inner lumen. The endometrium is a multi-cellular, dynamic tissue comprised of a basal layer adjacent to the myometrium and a functional layer on the luminal aspect (Fig. 1). It is the functional layer of the endometrium that is most responsive to the ovarian hormones, undergoing decidualization and menstrual breakdown and repair (proliferation). The endometrium has a columnar luminal epithelium covering a multi-cellular stromal compartment. The endometrial stroma is made up of fibroblast-like stromal cells, tissue-resident endometrial immune cells, and a varying influx of innate immune cells across the menstrual cycle (Critchley *et al.*, 2020a). The endometrium is supplied by a complex blood and lymphatic vasculature, including the specialized spiral arterioles (Rogers, 1996; Rogers *et al.*, 2008). It is the spiral arterioles that are invaded by trophoblast during the early stages of pregnancy (Lyall *et al.*, 2001).

For the most part, E_2_ and P_4_ exert their effects on the endometrium via their respective receptors, ER and PR-A and -B (PR-A or PR-B) (Fig. 1). Two separate ER isoforms have been identified; ER alpha (ERα) and ER beta (ERβ) (Couse *et al.*, 1997; Critchley *et al.*, 2001; Pettersson and Gustafsson, 2001; Gibson and Saunders, 2012). These receptors, along with receptors for androgens (AR), glucocorticoids (GR), and mineralocorticoids (MR), belong to a superfamily of nuclear receptors, which act as ligand-activated transcription factors (Patel *et al.*, 2015). ERα and ERβ are both expressed in the glandular epithelium and the stroma during the proliferative phase, which is when E_2_ induces endometrial cell proliferation. During the secretory phase, the levels of ERα decline in both the endometrial glandular epithelium and stroma. Although ERβ levels also decline in the glandular epithelium in the secretory phase, these receptors are maintained in the endometrial stroma (Critchley and Saunders, 2009). ERα and ERβ have structurally distinct ligand binding and amino-terminal A/B domains; therefore, the oestrogen response element (ERE)-dependent signalling pathways differ substantially between the two receptors. ERα demonstrates higher affinity for ERE binding and higher transcriptional activity in response to E_2_, relative to ERβ (Yu *et al.*, 2022).

Of the two PR isoforms, PR-A is expressed more dominantly and is an inhibitor of PR-B (Kastner *et al.*, 1990). Uterine PR expression is stimulated by E_2_ via ERα; hence, an appropriate P_4_ response is dependent on the presence of an adequate E_2_ response (Kowalik *et al.*, 2013). PRs are located in the nuclei of endometrial epithelial and stromal cells during the proliferative phase. During the secretory phase, PR-A is absent from glandular cells but persists in the stromal compartment of the functional layer of the endometrium, particularly in the perivascular region (Mote *et al.*, 2001; Critchley *et al.*, 2020b). PR-A is primarily responsible for endometrial function during decidualization; signalling between P_4_ and PR-A regulates molecular crosstalk between the luminal epithelium and stroma (Hirota, 2019). The relaxin receptor (RXFP1) is expressed in the endometrium, but there are conflicting reports as to its cellular localization and pattern of expression across the cycle; however, studies consistently report a role for relaxin and RXFP1 in the secretory phase and early pregnancy (reviewed by Marshall *et al.* (2017)).

It is now recognized that androgens also impact the proliferation, migration, and survival of stromal cells within human endometrium and may influence post-menstrual endometrial repair (Marshall *et al.*, 2011; Cousins *et al.*, 2016a). Endometrial AR expression varies throughout the menstrual cycle. AR is predominantly expressed in the stromal compartment of the functional endometrium during the proliferative phase, with reduced expression in the secretory endometrium. Glandular AR expression has been detected in the late secretory and decidualized endometrium (Milne *et al.*, 2005). In addition, there is the potential for effects of glucocorticoids via endometrial GR expression, as well as the recognized effects via the hypothalamic–pituitary–gonadal (HPG) axis, with recent research suggesting direct effects on endometrial stem cell function (Park *et al.*, 2021); of interest, are the potential direct effects of adrenal hormone production (via a stress response) on endometrial cyclicity, particularly during the menstrual phase.

Decidualization is the spontaneous differentiation and remodelling of uterine endometrial stroma that begins in response to P_4_ during the secretory phase in preparation for possible implantation (Gellersen and Brosens, 2014). Decidualization is critical to accommodate and moderate the extent of blastocyst implantation and trophoblast invasion during placentation (Murata *et al.*, 2022). Impaired decidualization is associated with many disorders of pregnancy including recurrent pregnancy loss (Lucas *et al.*, 2020) and preeclampsia (Founds *et al.*, 2009). *In vivo* and *in vitro* studies suggest that decidual cells can sense embryo quality to facilitate maternal rejection of incompetent embryos (Teklenburg *et al.*, 2010) and also act to promote trophoblast invasion (Menkhorst *et al.*, 2009, 2019). P_4_-stimulated production of cAMP in the endometrial stroma results in a change of cellular morphology from an elongated to a rounded appearance and induction of locally produced P_4_-dependent proteins, which enhance decidualization (e.g. prolactin, insulin-like growth factor binding protein 1, forkhead box protein O1, relaxin, Scribble, leukaemia inhibitory factor (LIF) and IL11) (Tabanelli *et al.*, 1992; Frank *et al.*, 1994; Kajihara *et al.*, 2013; Gellersen and Brosens, 2014; Altmäe *et al.*, 2017; Wang *et al.*, 2020; Critchley *et al.*, 2020a). Uterine resident leukocytes are also critical for decidualization; uNK, mast cells, T cells, and dendritic cells are critical for decidual angiogenesis (Evans *et al.*, 2016) and murine uNK and dendritic ablation cell models show these cells are essential for decidual integrity (Ashkar *et al.*, 2000; Plaks *et al.*, 2008).

The endometrium is receptive to blastocyst implantation only for a short period (window of implantation; WOI) during each menstrual cycle (Days 19–23 in a 28-day cycle, Fig. 1) (Evans *et al.*, 2016; Governini *et al.*, 2021). To achieve receptivity, the luminal epithelium undergoes significant differentiation, transforming from a non-adhesive surface to an adhesive surface, including focal enzymatic clearance of cell surface glycocalyx, epithelial–mesenchymal transition (EMT), and loss of epithelial polarity, tight junctions, and latero-junctional complexes. The glandular epithelium also becomes highly secretory during the WOI (Evans *et al.*, 2016). The importance of uterine glandular secretions in embryo implantation is highlighted by the infertility of the sheep uterine gland knockout model (Spencer and Gray, 2006) and murine knockout and pharmacological intervention models of glandular epithelium or specific glandular epithelial secreted factors including LIF (Stewart *et al.*, 1992; White *et al.*, 2007; Filant and Spencer, 2013).

In the absence of embryo implantation, P_4_ and E_2_ levels fall dramatically during the late secretory phase of the menstrual cycle (Fig. 1). The withdrawal of P_4_ in the presence of a decidualized endometrium results in an influx of inflammatory cells and mediators, vasoconstriction of the specialized spiral arterioles, an increase in matrix metalloproteinases, and breakdown and shedding of the functional layer of the endometrium at menstruation (Gellersen and Brosens, 2014; Jain *et al.*, 2022). The menstrual endometrium is shed in a piecemeal fashion, with endometrial repair commencing in areas adjacent to active breakdown (Garry *et al.*, 2009). Frequency of menstruation is typically every 24–38 days, for a duration of up to 8 days, with a volume of monthly loss between 5 and 80 ml (Fraser *et al.*, 2011; Munro *et al.*, 2018). For menstrual cessation, restoration of the luminal epithelial barrier is required, alongside appropriate endometrial haemostasis, repair of the damaged vasculature and subsequent stromal expansion (Ludwig and Spornitz, 1991). Vasoconstriction of endometrial vessels and the resulting endometrial hypoxia may be necessary to limit menstrual blood loss and ensure timely repair of the denuded menstrual endometrium (Markee, 1940; Fan *et al.*, 2008; Cousins *et al.*, 2016b; Maybin *et al.*, 2018). Hypoxia-inducible factor 1-alpha (HIF-1A) may be involved in hypoxia-regulated repair as levels have been shown to increase following P_4_ withdrawal at menstruation (Maybin *et al.*, 2018; Zhang *et al.*, 2022). By Day 5, vessel repair is normally complete and menstrual bleeding concludes (Maybin and Critchley, 2015). This cessation of bleeding marks commencement of the proliferative phase, where E_2_ is the dominant hormone acting on the endometrium (Fig. 1). During the proliferative phase, the functional layer of the endometrium grows rapidly, necessitating angiogenesis to support growth of new tissue (Girling and Rogers, 2005).

### Myometrium

The myometrium is the muscular central layer of the uterus, composed of outer longitudinal and sub-endometrial circular-orientated, junctional smooth muscle fibres, alongside supporting vasculature. Uterine myocytes show cyclical changes in steroid receptor expression (Fig. 1), with ER expression maximal in the proliferative phase, concentrated in the sub-endometrial myocytes, and declining sharply in the early secretory phase (Snijders *et al.*, 1992; Noe *et al.*, 1999). In contrast, PR expression appears to be consistent throughout the cycle (Snijders *et al.*, 1992; Noe *et al.*, 1999).

Throughout the menstrual cycle, the myometrium exerts wave-like contractions, called uterine peristalsis, which are thought to aid in sperm transit and embryo transport (Kunz and Leyendecker, 2002; Hunt *et al.*, 2020; Qu *et al.*, 2021). Myometrial contractions display cyclicity, increasing in strength and frequency during the proliferative phase to be maximal at ovulation and then retrograde (from the cervix to fundus), supporting sperm transit from the cervix to the uterine tube. In contrast, in the secretory phase, the myometrium is relatively quiescent, with low amplitude and bi-directional contractions, likely aiding apposition of the embryo to a suitable region of the endometrium (Hunt *et al.*, 2020). During menstruation, myometrial contraction is antegrade (fundus to cervix) to facilitate the expulsion of menstrual debris. In addition, although their frequency remains low, the amplitude of myometrial contractions increases during menstruation (Aguilar and Mitchell, 2010).

### Cervix

The cervix is the lower portion of the uterus that connects the body of the uterus and the vagina. Its main functions are to prevent entry of pathogens into the uterus from the vagina, to permit ascent of sperm, and to maintain a pregnancy in the uterus (reviewed by Martyn *et al.* (2014)). Structurally, the cervix is composed of the ectocervix (the portion projecting into the vagina) and endocervix (joining the vagina to the uterus). The endocervical canal is the space between the external os (opening of the endocervical canal) and the internal os, which opens into the uterine cavity. The ectocervix is comprised of stratified squamous epithelium, whereas the endocervix is lined with mucin-secreting columnar (glandular) epithelium (Fig. 1). At the squamocolumnar junction, where the ectocervix and endocervix join, columnar cells of the endocervix undergo metaplasia to the squamous cells of the ectocervix. The stroma of the cervix is comprised of fibro-muscular tissue densely made up of connective tissues, smooth muscle, and elastic tissues. The length and shape of the cervix are varied, with factors including age, parity, and hormonal changes associated with structural changes (Martyn *et al.*, 2014).

Cervical mucous fills the opening of the cervical canal and is composed of large polymeric glycosylated proteins called mucins, with MUC4 and MUC5B being the most abundant (Han *et al.*, 2017). The function of cervical mucous is associated with changes across the menstrual cycle that are critical for fertility (Martyn *et al.*, 2014). Mucin-secreting columnar epithelium are E_2_ and P_4_ responsive (Fig. 1). In association with rising E_2_ levels prior to ovulation, mucous secretion increases and becomes less viscous, which facilitates sperm migration. After ovulation, in the presence of increasing P_4_, mucous secretion drops and viscosity increases. Levels of various immune factors in cervical mucous display cyclical fluctuations, reducing at mid-cycle and then rising again towards the end of the cycle, mirroring E_2_ patterns (Han *et al.*, 2017). Interestingly, LIF, which is considered an important marker of receptivity in the endometrium, also shows cyclic expression in the cervix, becoming highest during the mid-secretory phase (Fukui *et al.*, 2022); however, its function in the cervix is unknown.

## Reproductive tract abnormalities caused, linked, or exacerbated by cyclic pathways

In the following section, we will detail abnormalities of the reproductive tract that are caused by, or exacerbated by, menstrual cycle changes. As above, discussion will be limited to the uterine tube, endometrium, myometrium, and cervix (in that order). Some conditions may impact more than one structure and some structures have been more frequently researched than others; we highlight the under-researched areas throughout. The perturbed physiology underpinning each of these conditions or symptoms must be determined if we are to increase our understanding of specific pathologies and facilitate the development of more precise treatments.

### Uterine tube

#### Ectopic pregnancy

Ectopic pregnancy accounts for around 1–2% of all pregnancies in Europe and the USA, the vast majority of which occur when implantation occurs erroneously within the uterine tube (Panelli *et al.*, 2015). Altered motility of the conceptus through the uterine tube in ectopic pregnancy may be explained by changes in smooth muscle and cilial function, with reduced tubal cilia found in women experiencing ectopic pregnancy (Vasquez *et al.*, 1983). An altered response to sex steroid regulation may play a role in ectopic pregnancy, with a lack of ERα found in the uterine tubes of women with ectopic pregnancy and, significantly, a reduction in PR expression (Horne *et al.*, 2009). In contrast, a high circulating level of P_4_ reduces murine oviduct ciliation and ciliary beat frequency (Bylander *et al.*, 2010). Artificial reproduction techniques are associated with ectopic pregnancy; with higher ectopic rates seen amongst pregnancies following fresh embryo transfers compared to frozen transfers, potentially resulting from altered tubal function in the presence of hyper-physiological circulating hormones post-ovarian stimulation (Perkins *et al.*, 2015). Further research is required to understand the mechanisms leading to disturbed conceptus motility, especially in women with no known risk-factors. This may enable the development of improved early pregnancy tests that can identify ectopic pregnancies, which are currently diagnosed by ultrasound.

#### Endometriosis

Endometriosis is an E_2_-dependent, chronic, systemic inflammatory condition caused by endometrial-like lesions that grow on and invade the surface of pelvic organs. Endometriosis can have deleterious consequences on the function of the uterine tube due to distorted tubo-ovarian anatomy and hematosalpinx. A recent meta-analysis reported possible evidence of an association between endometriosis and ectopic pregnancy (odds ratio = 2.16–2.66) (Yong *et al.*, 2020). The inflammation and adhesions associated with endometriosis can impact tubal function by decreasing tube motility, therefore negatively impacting transport of the oocyte and/or embryo (Macer and Taylor, 2012). Furthermore, some studies have identified an increased number of macrophages (including foam cells and haemosiderin-laden cells) in the uterine tubes of patients with endometriosis compared to controls (Matsushima *et al.*, 2002). Interestingly, one case-study of a patient seeking fertility treatment with a history of endometriosis reported hydrosalpinx of the left uterine tube with menstrual cycle-dependent changes in the size or volume of the tube, as indicated by ultrasound imaging (Osuga *et al.*, 2008).

### Endometrium

Discussed below are abnormal uterine bleeding, pelvic pain (or dysmenorrhoea), and infertility, which are the most common endometrial symptoms associated with patterns of aberrant cycling or lead to abnormal cycling.

#### Abnormal uterine bleeding

Abnormal uterine bleeding (AUB) may be due to a change in menstrual bleeding duration, frequency, regularity, and/or volume (Munro *et al.*, 2018). The underlying diagnosis may be structural or non-structural (with normal uterine anatomy). Structural pathology specifically affecting the endometrium includes abnormal uterine bleeding either due to endometrial polyps (AUB-P), of endometrial origin (AUB-E), or due to endometrial malignancy (AUB-M). Endometrial hyperplasia and endometrial cancer have been extensively reviewed elsewhere (Pennant *et al.*, 2017; Lu and Broaddus, 2020; Petersdorf *et al.*, 2022) and are not discussed further in this review.

Endometrial polyps are growths composed of endometrial glands, stroma, blood vessels, and fibrous connective tissue (Munro, 2019). They can form anywhere in the uterine cavity in single or multiple numbers, either pedunculated (elongated stalk of tissue) or sessile (dome-shaped), and can vary considerably in size (Munro, 2019; Nijkang *et al.*, 2019). Some polyps may spontaneously regress (Yuksel *et al.*, 2021), but symptomatic polyps often require surgical removal in the form of hysteroscopic polypectomy (American Association of Gynecologic Laparoscopists (AAGL), 2012; Nijkang *et al.*, 2019). The underlying mechanisms responsible for polyp development remain unknown; however, these benign growths have been linked to E_2_ cyclicity and differential ER and PR expression (Nijkang *et al.*, 2019; reviewed by Kossaï and Penault-Llorca (2020)). ER expression has been shown to be increased in polyps relative to normal endometrium, whereas others have reported a reduction in PR expression in polyps (Taylor *et al.*, 2003; Peng *et al.*, 2009; Peres *et al.*, 2018), and some studies have demonstrated no change in ER or PR expression. Studies have shown proliferative and secretory phase-dependent changes in both ER and PR expression in endometrial polyps (Taylor *et al.*, 2003; Peng *et al.*, 2009) and expression differences between premenopausal and postmenopausal women (McGurgan *et al.*, 2006; Gul *et al.*, 2010); this switch may explain the higher incidence of endometrial polyps observed following menopause (Nijkang *et al.*, 2019). Use of tamoxifen (a selective ER modulator) post-menopause increases the incidence of endometrial polyps compared to that seen in non-tamoxifen users (Deligdisch *et al.*, 2000). Polyps from tamoxifen users have elevated ER and PR expression compared to atrophic postmenopausal endometrium, and higher PR expression compared to the polyps from tamoxifen non-users (Leão *et al.*, 2013). Given the high prevalence of endometrial polyps (ranging from 7.5% to 50%) and high levels of recurrence following surgical removal (Yang *et al.*, 2015), it is essential that the hormone-based mechanisms of polyp growth are investigated to advance therapeutic and preventative management options.

AUB-E is the result of aberrant menstrual breakdown and repair leading to heavy menstrual bleeding (HMB). The inflammation, haemostatic mechanisms, vascular function, and tissue repair that occur during menstruation are tightly regulated to limit menstrual blood loss (Jain *et al.*, 2022). Patients with HMB have higher levels of TNFA protein in their menstrual effluent when compared to those with normal menstrual blood loss (Malik *et al.*, 2006). The prostaglandin (PG) synthesis pathway has also been shown to be overactive in those with AUB-E, with cyclo-oxygenase enzyme expression (*COX-1*/*PTGS1* and *COX-2*/*PTGS2*) and PG signalling higher in endometrium from those with objectively measured HMB versus those with normal menstrual blood loss (Smith *et al.*, 2007). This increased inflammatory response in those experiencing AUB-E explains the clinical effectiveness of non-steroidal anti-inflammatory medications during menstruation as a treatment for HMB (Cameron *et al.*, 1990; Bofill Rodriguez *et al.*, 2019); however, the underlying reason for the differential inflammatory response remains unknown.

The coagulation system is central in the limitation of blood loss. Disorders that affect systemic haemostasis will impact menstrual blood loss; e.g. von Willebrand disease has a prevalence of 13% in those presenting with HMB relative to the general population (Shankar *et al.*, 2004). Specific endometrial haemostatic defects may also contribute to the symptom of HMB, with evidence of over activation of the fibrinolytic system in those with AUB-E (Gleeson *et al.*, 1993). This provides a mechanism for the effectiveness of tranexamic acid as a treatment for HMB (Gleeson *et al.*, 1994).

The specialized endometrial vessels are remodelled during the secretory phase, becoming heavily coiled spiral arterioles that lack an internal elastic lamina (Robertson and Manning, 1974). Evidence for pre-menstrual vasoconstriction of these arterioles was first demonstrated with direct visualization of endometrial explants transplanted into the anterior eye chamber of the Rhesus monkey (Markee, 1940). As the radius of a vessel is the major determinant of resistance to flow, it is likely that this intense vasoconstriction limits menstrual blood loss. Those experiencing AUB-E had lower levels of vasoconstrictors in their endometrium (Marsh *et al.*, 1997; Smith *et al.*, 2007), consistent with inefficient spiral arteriole vasoconstriction during menses. In addition, those experiencing HMB were shown to have larger endometrial vessel wall circumference, more focal discontinuities (Mints *et al.*, 2007), reduced vascular smooth muscle cell proliferation, and lower markers of vessel maturity during the secretory phase (Abberton *et al.*, 1999a,b). Furthermore, components of the endometrial endothelial extracellular matrix (laminin (LAM), osteopontin (SPP1), fibronectin (FN1), and collagen IV (COL4)) are dysregulated in AUB-E and may contribute to reduced endothelial vascular integrity (Biswas Shivhare *et al.*, 2018). Hence, aberrant vascular remodelling and/or decreased endometrial vasoactive factors may serve to prevent the necessary vasoconstriction of endometrial vessels at menstruation which normally limits menstrual blood loss.

A lack of endometrial vessel vasoconstriction at menstruation may also result in a lack of physiological endometrial hypoxia during menstruation and impact subsequent repair. HIF1A is the master regulator of the cellular response to hypoxia and initiates the transcription of a host of downstream targets involved in angiogenesis, metabolism and tissue remodelling (Semenza, 2000). Patients with AUB-E had decreased menstrual endometrial HIF1A protein and its downstream targets when compared to those with normal menstrual bleeding (Maybin *et al.*, 2018). In a mouse model of simulated menses, genetic or pharmacological reduction in HIF1A delayed endometrial repair (Maybin *et al.*, 2018). Other putative endometrial repair factors, e.g. TGFB, have also been detected at lower levels in the premenstrual endometrium of those with HMB (Maybin *et al.*, 2017). Lower menstrual levels of HIF1A and TGFB in patients with AUB-E may affect vascular repair, angiogenesis, and restoration of the luminal epithelial barrier after endometrial shedding (Lu *et al.*, 2021; Watters *et al.*, 2022). Further research to increase the underlying cause of AUB in those without structural abnormalities will increase precision in the treatment of this debilitating symptom.

#### Pelvic pain: endometriosis and secondary dysmenorrhoea

The most predominant symptom of endometriosis is pain. Endometriosis-associated pain is as complex as the disease itself with various types of pain recognized to be involved (nociceptive pain including inflammatory pain, neuropathic pain, and centralized pain) (Morotti *et al.*, 2017; Coxon *et al.*, 2018). Furthermore, some individuals may experience menstrual cycle-associated exacerbations in pain (severe dysmenorrhoea or ovulation pain), or constant non-cyclical pelvic pain, or pain that is function-dependent (dyschezia, dysuria, or dyspareunia), or a different combination of any of these (Morotti *et al.*, 2017).

As the topic of this review is cyclical processes of the reproductive tract and how these cyclic processes link with pathology, we will focus on endometriosis-associated secondary dysmenorrhoea (see myometrial section below for discussion of primary dysmenorrhoea). Endometriosis is the most common cause of secondary dysmenorrhoea (Clemenza *et al.*, 2021). Those who report experiencing dysmenorrhoea are 2.6 times more likely to have endometriosis compared to those who report no (or infrequent) dysmenorrhoea (Treloar *et al.*, 2010). Those with endometriosis have more severe menstrual pain, and also report that their menstrual symptoms have more impact on their lives compared to those without endometriosis (Evans *et al.*, 2021).

The menstrual phase of the cycle is associated with increased endometrial PG levels, which are synthesized by COX-1 and -2 from cellular phospholipid membranes released during endometrial breakdown (Jabbour *et al.*, 2006; Oladosu *et al.*, 2018). PGs play an important role in uterine contractility and dysmenorrhoea. E_2_ and P_4_ are physiological regulators of PG production and secretion in the endometrium (Salamonsen and Findlay, 1990). Both COX-2 and PGs (PGE_2_, PGF_2a_) have been found to be elevated in endometrium and endometriotic lesions of patients with endometriosis relative to controls (Jabbour *et al.*, 2006; Oladosu *et al.*, 2018). PGE_2_ plays a significant role in the development and maintenance of endometriosis. In response to PGE_2_, there is evidence for endometriotic stromal cell expression of both steroidogenic acute regulatory protein (StAR) and cytochrome P450 aromatase (CYP19), which results in aberrant production of E_2_, thus enhancing a positive feedback loop (Wu *et al.*, 2007). Therefore, in the endometriosis setting, dysmenorrhoea may be more pronounced as a result of an E_2_ and PG imbalance, not apparent in those without endometriosis.

#### Infertility: implantation failure

Synchrony between the competent blastocyst and receptive endometrium is essential for successful implantation. Prior to implantation, the blastocyst ‘floats’ inside the uterine lumen for up to 72 h (Norwitz *et al.*, 2001). It is thought that, during this period, secreted factors from the blastocyst and uterine epithelium act on each other to promote receptivity and facilitate adhesion (Cuman *et al.*, 2013; Governini *et al.*, 2021). Studies in mice have identified many endometrial epithelial-produced factors where dysregulated production causes infertility (e.g. LIF (Stewart *et al.*, 1992), Jagged-1 (Zhou *et al.*, 2021; Gurner *et al.*, 2022)). The endometrial receptivity array (ERA) test aims to identify the WOI in those undergoing IVF, thus enabling the timing of embryo transfer to be adjusted (Díaz-Gimeno *et al.*, 2013). Meta-analyses of and a retrospective observational study of ERA efficacy have identified that there is no benefit to ERA testing during IVF (Liu *et al.*, 2022; Cozzolino *et al.*, 2022a; Arian *et al.*, 2023), possibly because whole endometrial biopsies, containing variable epithelial, stromal, endothelial, and immune cell composition are used for ERA testing. This likely limits the ability of the test to detect single-factor, cell-specific changes that have been shown to impair implantation in animal and *in vitro* models. More research is required to identify diagnostic targets and suitable clinical samples capable of detecting cell-type-specific disturbance in these targets.

#### Infertility: thin endometrium

Endometrial thickness is routinely measured to assess endometrial receptivity. No consensus exists on the definition of thin endometrium (endometrial atrophy); however, it is often reported as <7 mm on the day of ovulation (von Wolff *et al.*, 2018; Liao *et al.*, 2021; Xu *et al.*, 2022). Thin endometrium is implicated in lower pregnancy rates and adverse perinatal and maternal outcomes (Liao *et al.*, 2021; Xu *et al.*, 2022). The mechanism(s) causing thin endometrium remains unknown, and likely include inflammation and iatrogenic damage, with limited treatment options available (Xu *et al.*, 2022). High oxygen levels associated with the basalis layer have been hypothesized to lead to the presence of damaging reactive oxygen species, an unfavourable environment compared to the normally low oxygen tension of the endometrial surface (Revel, 2012). More research is needed to determine the clinical utility of endometrial thickness, the causal factors, and the impact of a thin endometrium on receptivity and implantation rates.

#### Infertility: recurrent pregnancy loss

The spontaneous decidualization that initiates regardless of implantation is crucial for a healthy pregnancy. Following implantation, decidualization continues to create the decidua of pregnancy (Dimitriadis *et al.*, 2010), driven by locally released factors from the decidual cells, decidual immune cells and invasive trophoblast that secrete P_4_ and profilin-1 to promote decidualization (Genbacev *et al.*, 1992; Menkhorst *et al.*, 2017). Women with recurrent pregnancy loss show depleted decidual cell precursors including endometrial mesenchymal stem-like cells (Lucas *et al.*, 2016), and proliferative mesenchymal cells (Diniz-da-Costa *et al.*, 2021). This depletion results in excessive decidual senescence (Lucas *et al.*, 2020), suggesting that the endometrium lacks plasticity to facilitate decidual expansion during pregnancy (Dimitriadis *et al.*, 2020).

The decidua is also proposed to ‘sense’ embryo quality and block implantation and development of non-viable embryos (Teklenburg *et al.*, 2010; Brosens *et al.*, 2014; Macklon and Brosens, 2014). Numerous studies have demonstrated that P_4_ actions on the endometrial luminal epithelium are mediated via the stroma (Evans *et al.*, 2016). In women with recurrent miscarriage, the luminal epithelium has altered expression of factors that regulate blastocyst adhesion, such as integrins (Quenby *et al.*, 2007), galectin-7 (Menkhorst *et al.*, 2014), and MUC16 (Liu *et al.*, 2020). These findings have led to speculation that poor quality blastocysts are able to abnormally adhere to the luminal epithelium, initiating implantation and a pregnancy that must ultimately fail (Quenby *et al.*, 2007).

#### Infertility: endometriosis

Endometriotic lesions and adhesions can radically distort the pelvic anatomy, negatively impacting fertility (for example impeding oocyte release, altering sperm motility, impairing embryo transport, and altering myometrial contractions) (Macer and Taylor, 2012; Nicolaus *et al.*, 2020). Aside from these mechanical disturbances, the altered hormone and inflammatory milieu of endometriosis also disrupts the physiology of the endometrium and uterine tubes (as further discussed in ‘uterine tube’ section above).

Recently, single-cell analyses performed on endometrial biopsies of patients with and without endometriosis demonstrated significant differences in the abundance of immune cells, with differences that were exacerbated by cycle stage (Vallve-Juanico *et al.*, 2022). In association with endometriosis, there was an increase in endometrial macrophages and neutrophils (thus greater inflammatory phenotype) during the proliferative phase, but endometrial CD91^+^ macrophages and CD1a^+^ dendritic cells exhibited decreased phagocytic capacity (Vallve-Juanico *et al.*, 2022). As endometrial tissue immune populations are actively involved in pregnancy establishment, and endometrial immune cell dysfunction is augmented in endometriosis, there is an urgent need to increase knowledge of immune cell abundance and activity within the endometrium across the various stages of disease (compared with controls) in an effort to elucidate effects of endometriosis on pregnancy outcomes (Vallvé-Juanico *et al.*, 2019).

In the healthy endometrium, P_4_ and E_2_ tightly regulate the menstrual cycle to allow for implantation; however, in endometriosis, the balance between E_2_ and P_4_ is disrupted. Endometriosis is frequently described as a state of E_2_ dominance and P_4_ resistance. P_4_ resistance describes a malfunction of the endometrium (and ectopic endometrium) to respond appropriately to bioavailable P_4_, thus encompasses both failed activation of PR and failure of P_4_-targeted gene transcription (reviewed by Marquardt *et al.* (2019)). Not only does this P_4_-resistant environment favour E_2_-related inflammation and cell proliferation, it also leads to inadequate decidualization and a non-receptive endometrium (Marquardt *et al.*, 2019).

### Myometrium

It is becoming clear that disorders of the myometrium, including primary dysmenorrhoea, uterine fibroids, and adenomyosis, can negatively impact menstrual symptoms and fertility (Bourdon *et al.*, 2020; Qu *et al.*, 2021; Vrijdaghs *et al.*, 2022). Despite their undisputed impact on quality of life, studies recognizing the central role of myometrium in these diseases are still under-represented in the research literature.

#### Primary dysmenorrhoea

Primary dysmenorrhoea is the most common gynaecological symptom in reproductive-aged women, with the literature reporting a range that varies between 24% and 92% (Morrow and Naumburg, 2009; Wang *et al.*, 2022). A recent meta-analysis of primary dysmenorrhoea among students found an overall prevalence of 66.1% based on almost 100 studies (including 78 068 participants) (Wang *et al.*, 2022). There may be wide variation in the degree of symptomatology from person to person, and also from cycle to cycle in individuals (Morrow and Naumburg, 2009). Symptoms commonly start within hours of menstrual flow beginning and can last for up to 72 h (Dawood, 1990; Morrow and Naumburg, 2009). Pain occurs in the lower abdominal or suprapubic area and is described as crampy and diminishing in intensity, and may also be felt in the lower back and inner thighs. As already described, the pathogenesis of dysmenorrhoea is associated with the overproduction of uterine PGs under the regulation of E_2_ and P_4_ (Salamonsen and Findlay, 1990; Jabbour *et al.*, 2006; Oladosu *et al.*, 2018). Therefore, the most commonly used treatments for primary dysmenorrhoea are non-steroidal anti-inflammatory drugs (NSAIDs), which are frequently very effective (Marjoribanks *et al.*, 2015). However, up to 25% of people do not respond to NSAIDs or suffer intolerable side-effects; for these people, oral contraceptives (OCs) that suppress ovulation and reduce serum E_2_ and P_4_, leading to decreased production of PGs and supposedly pain, are frequently employed (Wang *et al.*, 2022). However, a Cochrane review demonstrated that there was insufficient evidence to support the position that OCs were effective in treating primary dysmenorrhoea (Proctor *et al.*, 2001). Given the high prevalence of primary dysmenorrhoea and its significant negative impact on quality of life, there is a pressing need for further, high-quality studies to understand underlying mechanisms and to determine treatment effectiveness in this area.

#### Uterine fibroids

Uterine fibroids (or leiomyoma) are common benign tumours of the myometrium. The prevalence of uterine fibroids is high, found in 60–80% of women during their lifetime (symptomatic in 20–50%) (Owen and Armstrong, 2015; Navarro *et al.*, 2021). Fibroid-associated symptoms include HMB with subsequent anaemia, subfertility, pelvic pressure and pain, urinary incontinence, and dyspareunia. Within the uterus, fibroids can develop in multiple locations including submucosal, intramural, subserosal, and may also be pedunculated (intracavitary or subserosal).

Mechanisms of uterine fibroid development and growth are multifactorial (Al-Hendy *et al.*, 2017); however, these tumours frequently regress after menopause, suggestive of P_4_ and E_2_ sensitivity. *PRB* and total *PR* mRNA are higher in cells derived from uterine fibroids compared to the myometrium (Holdsworth-Carson *et al.*, 2014). Furthermore, E_2_ and ER are higher in uterine fibroids relative to normal myometrium (Kovács *et al.*, 2001). SMA and proliferating cell nuclear antigen (PCNA) immunostaining are also higher in fibroids compared with myometrium; this increase is associated with the proliferative phase of the menstrual cycle (Holdsworth-Carson *et al.*, 2016). In contrast, fibroid PCNA levels drop during menopause (Holdsworth-Carson *et al.*, 2016). A recent longitudinal study found that fibroids have a median 6-month growth rate of 13% in postmenopausal patients, compared to an annual average growth rate of 18–82% in premenopausal women (Shen *et al.*, 2021).

The presence of uterine fibroids also alters endometrial cyclic production of numerous factors important in regulating endometrial receptivity and decidualization (including HOXA10/11, LIF, IL11, and TGFβ3) (Navarro *et al.*, 2021). Fibroids may also lead to AUB, with some evidence of impaired vasoconstriction at the time of menstruation in women with fibroids, with fibroid tissue expressing altered levels of endothelin receptors and PGF_2α_ when compared to normal myometrium (Pekonen *et al.*, 1994; Miura *et al.*, 2006). This could significantly increase menstrual blood volume.

Non-surgical, pharmacologic therapies for uterine fibroids include treatments modalities that largely focus on improving fibroid symptoms by reducing the cyclic activity of steroid hormones (Owen and Armstrong, 2015). Relugolix is an oral GnRH antagonist that has been recently demonstrated to provide significant improvement to a number of fibroid-related symptoms including menstrual blood loss, pain, anaemia, and uterine volume; it can be given in combination with oestradiol and progestogen, which mitigates risk of bone density loss and some of the menopausal symptoms associated with GnRH agents (Al-Hendy *et al.*, 2021; Baird and Harmon, 2021). Ulipristal acetate (UPA) is a selective PR modulator with mixed agonist and antagonist effects. It has been demonstrated in multiple clinical trials to be an effective treatment for HMB secondary to fibroids (Donnez *et al.*, 2012a,b, 2014, 2015; Illingworth *et al.*, 2018; Simon *et al.*, 2018) but has recently lost popularity due to concerns about drug-induced liver injury, with the risk estimated at 11 in 100 000 (Middelkoop *et al.*, 2021).

#### Adenomyosis

Adenomyosis is a condition of the myometrium, defined by the presence of ectopic nests of endometrial glands and stroma surrounded by reactive smooth muscle hyperplasia (recently reviewed by Guo (2022), Moawad *et al.* (2022), and Cozzolino *et al.* (2022b)). Adenomyosis is characterized by pain (dysmenorrhoea, dyspareunia, and chronic pelvic pain), AUB, and subfertility. The mechanism by which adenomyosis causes subfertility is not fully understood, but women with adenomyosis show impaired decidualization (Jiang *et al.*, 2016), including reduced expression of Scribble (Peng *et al.*, 2021). The underlying mechanisms resulting in AUB are equally ill-defined. *In vitro* studies examining cells from adenomyotic lesions reveal higher mRNA concentrations of prostaglandin E synthase 2 (*PTGES2*), the enzyme responsible for PGE2 synthesis, compared to eutopic endometrial cells from those without adenomyosis (Chen *et al.*, 2010). Adenomyoic lesions display extensive tissue fibrosis which has been quantified by elastosonography, an ultrasonic measurement of tissue stiffness, and found to positively correlate to volume of menstrual blood loss (Huang *et al.*, 2022). In the same study, fibrotic adenomyotic lesions expressed lower levels of HIF-1A, COX-2, EP2 and EP4, suggesting the lack of the key mediators in limitation of menstrual blood loss as described above in the AUB section (Huang *et al.*, 2022). However, studies comparing eutopic endometrial function in those with and without adenomyosis are required to better delineate the causes of AUB. A mouse model of adenomyosis had been created by inducing mechanical injury to the endometrial–myometrial interface, resulting in ectopic infiltration of endometrial stromal cells into the myometrial layer; supporting the theory that adenomyosis may, in some cases, be caused iatrogenically by endometrial injury or disruption. Interestingly, the authors were able to reduce the incidence of adenomyosis by preadministration of the neurokinin receptor antagonist, aprepitant, where the receptor mediates downstream targets of Substance P and local inflammation hypothesized to play a role in adenomyosis and endometriosis pathophysiology (Hao *et al.*, 2020).

As adenomyosis is poorly understood, hysterectomy remains the gold standard for treatment (Rathinam *et al.*, 2022). Continuing improvements in imaging modalities for the initial diagnosis and early assessment of adenomyosis (by both transabdominal and transvaginal ultrasound or MRI) should facilitate research and lead to improved treatment options. Medical management of adenomyosis typically focuses on balancing symptom relief with the desire for pregnancy. A recent systematic review highlighted that LNG-IUS (levonorgestrel intrauterine system), dienogest, and GnRH analogues were effective in treating some of the symptoms of adenomyosis; however, evidence in this space is lacking and laden with issues (e.g. small studies, non-trial setting, short duration of intervention and limited long-term analysis) (Rathinam *et al.*, 2022).

### Cervix

A primary function of the cervix is to prevent infection, whereby cervical mucous provides both a physical and chemical barrier to ascending genital pathogens. Pattern recognition receptors (PRRs) identify pathogens and are sex hormone sensitive; E_2_ has been shown to down-regulate some key PRRs and downstream inflammatory mediators in the cervix (Wira *et al.*, 2015). E_2_ also down-regulates antimicrobial peptide production in the lower genital tract (Medina-Estrada *et al.*, 2018), with mouse models showing increased sensitivity to gonorrhoea infection with increased E_2_ (Kita *et al.*, 1985). High levels of E_2_ also increase epithelial attachment of *Chlamydia trachomatis* and *Neisseria gonorrhoea* (Maslow *et al.*, 1988). Use of combined OCs has been shown to increase risk of both chlamydia and gonorrhoea infection compared with that in non-users (Louv *et al.*, 1989). The higher infection rates detected amongst users of OC preparations containing higher androgenic progestin components suggests a role for the hormonal influence on local anti-microbial action, rather than potential differences in sexual behaviour amongst OC users (Louv *et al.*, 1989). Cervical mucous of OC users has been shown to have reduced anti-chlamydial action (Mahmoud *et al.*, 1994). Whether localized infection translates into clinically significant disease is less clear. Studies assessing impact of OC use on pelvic inflammatory disease (PID) have shown that OC use appears to reduce the risk of symptomatic chlamydial PID (Wølner-Hanssen *et al.*, 1990; Spinillo *et al.*, 1996). Studies examining the relationship between PID and OC are limited by appropriate comparator groups and difficulties associated with accurate diagnosis, particularly with less severe PID, meaning definitive answers on the role of cervical changes in PID risk remain elusive (reviewed by Washington *et al.* (1985)). Higher rates of PID in women with intrauterine contraceptive devices highlight the important barrier role of the cervix in the prevention of ascending infection (Eschenbach *et al.*, 1977). Following menopause, reduction in E_2_ causes localized atrophy and can cause stenosis of the cervical os, there is lack of protective cervical mucous, with associated enhanced localized inflammation and decreased local immunity. This enhances susceptibility to local infection and malignancy during menopause (Ghosh *et al.*, 2014).

## Conclusions

Although the ovaries are the main site of steroid hormone production, the effects of fluctuating steroid hormone levels associated with the menstrual cycle can be observed in the various anatomical structures of the reproductive tract (the uterine tubes, endometrium, myometrium, and cervix). Each organ performs discrete functions that are critical to prepare for an ensuing pregnancy. While the uterus has been quite heavily studied, the same cannot be said for other components of the reproductive tract. In the event of pathology, each of these structures plays a role in an assortment of poor symptomologies that can have debilitating and chronic impacts on quality of life. It is imperative that conversations about menstruation and female anatomy are normalized amongst healthcare, workplace, school, and home environments. Reducing the social stigma that surrounds menstrual bleeding and related symptoms will increase community understanding of menstrual health and enable dysfunction to be identified and diagnosed earlier.

### Research directions

Research encompassing menstrual health and non-cancerous gynaecology remain underfunded compared to other women’s health conditions (for example a PubMed search between 2012 and 2022 on ‘benign gynaecology’ led to 6300 publications and ‘menstruation’ resulted in 6400 publications compared to breast cancer, with >230 000 publications) and conditions that impact both genders (diabetes, >465 000 publications; or heart disease, >560 000 publications in PubMed 2012–2022). A positive flow on effect from increased awareness and advocacy for menstrual health will be increased lobbying to improve access and availability to research funding. This would not only result in more research but would also lead to the inclusion of more women (young, old and pregnant) in clinical trials, whereas they have previously been under-represented in clinical research practices relative to their male counterparts. Rectifying these inequalities and prioritizing research that fills the existing knowledge gaps in understanding the normal and abnormal physiological mechanisms of the female reproductive tract will advance reproductive and gynaecological treatments to the benefit of millions of women globally.

Further research is required to:

- better understand the subtle effects of genetic, epigenetic, and hormonal variations on development and, ultimately, function and cyclicity of the uterine tubes, endometrium, myometrium, and cervix; these variations will likely help explain the underlying pathophysiology linked to the spectrum of presentation and symptoms associated with diseases like endometriosis, adenomyosis, and uterine fibroids;
- improve understanding of the interactions of spermatozoa, ovulated ova, and the zygote with the mucosal lining of the uterine tubes; knowledge of these interactions will contribute to our understanding of the mechanisms of tubal infertility and ectopic pregnancy;
- increase knowledge of the complex interactions among traditional (i.e. E_2_, P_4_) and non-traditional female hormones (e.g. androgens, glucocorticoids), and their associated receptors (e.g. ERα, ERβ, PR-A, PR-B, AR, GR), involved in regulating reproductive tract function; a recognition of the heterogeneity of the hormonal milieu and its impact on tract function will improve our knowledge of tract pathophysiology;
- improve understanding of the mechanisms responsible for the breakdown and repair of endometrial tissues during menstruation; a better understanding of the impact of systemic physiology on the menstrual process and its variations is needed to better understand cycle-to-cycle variability including cases of AUB and the aetiology of endometriosis;
- better understand immune cell abundance and activity within the different tract regions (including endometriosis, fibroids, adenomyosis, and polyps) and the variability in activity associated with dysfunction;
- elucidate the mechanisms of vascular cyclicity and their impact on tract function and dysfunction that lead to AUB; studies should clearly differentiate lymphatic and blood vascular function and identify the interaction with tract immune cell populations;
- understand the impact of cyclicity and myometrial peristalsis on the functionality and relative distribution or proportion of muscle cells and extracellular matrix in the myometrium; ongoing research is required to determine the role of myometrium in dysmenorrhoea, uterine fibroids, and adenomyosis;
- better understand the mechanisms responsible for the development of uterine polyps and the associated risk of polyp presence on disease incidence;
- increase our knowledge of the mechanisms responsible for painful menstruation; research is required to understand the mechanisms that interact with endometrial and myometrial factors to worsen danger signals responsible for dysmenorrhoea.

## Data Availability

No new data were generated or analysed in support of this review.
